# Stigma of hearing loss and the use of hearing aids among adults with hearing loss: a cross-sectional study

**DOI:** 10.3389/fpubh.2026.1962320

**Published:** 2026-09-24

**Authors:** Rania Alkahtani, Samira Mahboub, Fatmah Almoayad

**Affiliations:** 1 Department of Health Communication Sciences, College of Health and Rehabilitation Sciences, Princess Nourah bint Abdulrahman University, Riyadh, Saudi Arabia; 2 Department of Health Sciences, College of Health and Rehabilitation Sciences, Princess Nourah bint Abdulrahman University, Riyadh, Saudi Arabia

**Keywords:** health care access, hearing aids, hearing loss, Saudi Arabia, stigma

## Abstract

**Objectives:**

This study explored stigma associated with hearing loss and hearing aid use among adults in Saudi Arabia. It examined the prevalence of hearing loss stigma (HLS) and hearing aid stigma (HAsS) and their associations with hearing aid use and selected demographic characteristics.

**Methods:**

A cross-sectional survey was conducted among 403 adults with self-reported hearing loss. Participants completed an online questionnaire, distributed through convenience sampling, that included validated instruments assessing HLS, HAsS, and personal distress/inadequacy related to hearing loss.

**Results:**

High levels of HLS and HAsS were reported by 50.12% and 82.93% of participants, respectively. Higher HLS levels were significantly associated with hearing aid use, lower educational attainments, unmarried status, lower incomes, greater self-reported hearing loss severity, and a longer duration of hearing loss. Notably, older individuals with lower incomes reported the highest levels of stigma. Among participants medically indicated for hearing aids, higher HAsS was associated with lower odds of hearing aid use, whereas higher HLS was associated with greater odds of use after adjustment for other model covariates. HLS was also strongly associated with personal distress and inadequacy.

**Conclusions:**

High levels of HLS and HAsS were observed within the study sample, highlighting stigma as a prominent psychosocial concern. HLS and HAsS showed different associations with hearing aid use, with higher HAsS associated with lower odds of use and higher HLS associated with greater odds of use. These findings highlight the potential importance of addressing stigma within hearing healthcare and support further investigation of approaches to address HL- and HA-related stigma in the Saudi context.

## 1 Introduction

Hearing loss (HL) is widely recognized for its detrimental effects on communication abilities, often leading to social isolation, feelings of inadequacy, depression, anxiety and other adverse health outcomes (1). In addition, HL can substantially affect employment opportunities (2). Given the multi-faceted impact of HL on individuals' lives, effective management requires a comprehensive approach that extends beyond restoring hearing alone.

Although the degree of HL directly influences communication abilities, successful rehabilitation is also shaped by personal factors, including communication needs, previous experiences, personality, self-perception, and coping strategies (3). Social factors, such as support from family, peers, and the broader community, further influence rehabilitation outcomes (4–6). Hearing aids (HAs) are a form of assistive technology designed to support hearing and communication in individuals with HL (7). However, stigma associated with HL and hearing aid (HA) use may discourage individuals from acknowledging their hearing difficulties, seeking professional help, and engaging with appropriate rehabilitation (8). Recent evidence further demonstrates that individuals may respond to HL and HA stigma by concealing their hearing difficulties or HA use (9). In many cases, denial of HL delays access to healthcare services, highlighting the importance of interventions that involve families and communities to reduce stigma and encourage help-seeking. Evidence suggests that community-based programs, photo-based campaigns, theory-driven interventions, and national screening programs can reduce stigma, improve self-efficacy, and promote help-seeking behaviors related to HL and HA use (10–13). Despite these known challenges, little is known about HL- and HA-related stigma in Saudi Arabia, where unique cultural and demographic characteristics may influence individuals' experiences of stigma (14, 15).

A meta-synthesis further demonstrated that although HAs improve communication, social participation, and emotional wellbeing, these benefits are frequently undermined by stigma, which remains an important barrier to HA uptake (16). These findings highlight the importance of adopting a holistic approach to HL management that addresses both the clinical and psychosocial aspects of hearing rehabilitation.

Previous studies have described different manifestations of stigma, including perceived stigma and self-stigmatization, with factors such as HA visibility contributing to these experiences, particularly among older adults (17, 18). Recent evidence has identified stigma associated with both HL and HAs, including perceptions related to aging, disability, and being different (19). In Saudi Arabia, older adults currently account for only 3.5% of the population; however, demographic projections indicate a substantial increase after 2030 as the current adult population ages (20). Early efforts to reduce HL stigma are therefore important not only to improve the current quality of life of individuals with HL but also to prepare for this demographic transition. At the same time, stigma may influence help-seeking behavior and HA acceptance across all age groups. Despite its potential importance, locally contextualized evidence on HL- and HA-related stigma in Saudi Arabia remains limited.

Conceptually, the present study considers stigma within a broader set of demographic and hearing-related factors that may shape hearing-health experiences and behaviors. Demographic characteristics, including age, sex, education, marital status, and income, together with hearing-related characteristics such as severity and duration of HL, may be associated with Hearing loss stigma (HLS) and Hearing aids stigma (HAsS). HLS and HAsS, in turn, may relate to psychosocial experiences, including Personal distress/inadequacy (PDI), and hearing-health behaviors, including seeking hearing assessment and HA use. These relationships are considered potentially interrelated rather than causal, given the cross-sectional design of the study. Therefore, this study aims to: (1) explore stigma associated with HL, (2) examine stigma toward HA use among adults with HL in Saudi Arabia, and (3) identify demographic and personal factors associated with both types of stigma. Understanding these factors may help inform the development of culturally appropriate counseling strategies, support services, and stigma-reduction interventions to improve the quality of life of individuals with HL.

## 2 Materials and methods

A cross-sectional study was conducted from September 2022 to April 2023. The design allowed for the collection of data at a specific point in time, providing a snapshot of the current state of HL and its associated stigmatization within the selected population. While this is appropriate for exploratory stigma-related research, the researchers acknowledge that this design limits causal inference and may be influenced by social desirability biases.

### 2.1 Participants and recruitment

Potential respondents included adults 18+ years old with self-reported HL who were residents of Saudi Arabia regardless of their gender, race and nationality. Participants were recruited using a convenience sampling approach between September 2022 and February 2023 via an online survey hosted on SmartSurvey, disseminated through X, WhatsApp, Telegram, and email. The survey invitation and questionnaire link were shared both publicly and within groups, and online accounts of hard-of-hearing organizations and groups were approached to facilitate dissemination. No specific hashtags were used. This approach was selected for its feasibility and ability to reach a geographically diverse population across Saudi Arabia. Because the survey was disseminated through multiple channels and could be further shared by recipients, the total number of individuals potentially reached could not be determined. In addition, data on views, impressions, link clicks, and the number of members in the groups through which the survey was distributed were not systematically recorded. Therefore, a recruitment response rate could not be calculated. This recruitment approach may have introduced sample bias, favoring individuals who are more socially connected and technologically literate, possibly underrepresenting more isolated individuals. To include older adults who were unable to read the questionnaire independently, relatives were permitted to read the questionnaire and record the participant's responses. Twelve participants (2.98% of the final sample) were illiterate and therefore required this assistance. No standardized instructions beyond reading the questions and recording the participant's responses were provided to assisting relatives.

The required sample size was calculated using a 95% confidence level and a 5% margin of error based on a Saudi disability survey estimating a population of approximately 60,000 individuals with hearing loss (21), yielding a minimum required sample size of 385 participants. An expected prevalence was not explicitly specified in the original calculation, and no design effect or additional allowance for nonresponse was applied. The sample-size calculation assumes random sampling; however, participants were recruited using convenience sampling. Therefore, the calculated sample size should be interpreted as a target for sample adequacy rather than as ensuring population representativeness. A total of 515 responses were collected, with no duplicate responses identified. After excluding 112 incomplete responses, the final analytical sample consisted of 403 participants (Figure 1).

**Figure 1:**
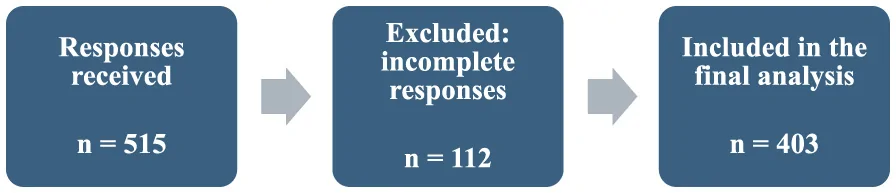
Participant flow diagram.

### 2.2 Materials and data collection

Data were collected using an Arabic translated version of the Hearing Attitudes in Rehabilitation Questionnaire (HARQ), which is a validated instrument designed to assess attitudes toward HL and HA use (22). The original tool consists of seven sections: (1) Personal distress/inadequacy (PDI), (2) Hearing loss stigma (HLS), (3) Minimization of hearing impairment, (4) Hearing aids stigma (HAsS), (5) Don't want/need aid, (6) Pressure to be assessed and (7) Positive expectation of HA. The present study utilized four of the seven HARQ subscales: PDI, HLS, HAsS, and Pressure to be Assessed. These subscales were selected *a priori* because they directly addressed the study objectives concerning stigma associated with HL and HA use and related psychosocial responses. The remaining subscales—Minimization of Hearing Impairment, Don't want/need aid, and Positive Expectation of Hearing Aid—assess constructs outside the primary focus of the present study and were therefore not included to reduce questionnaire length and participant burden. The original HARQ was developed as seven factorially derived subscales, with reliability and scoring evaluated separately for each subscale, supporting their interpretation as distinct attitudinal domains (22). HLS assesses stigma associated with having HL, whereas HAsS assesses stigma specifically associated with HAs and their use. PDI represents personal distress and feelings of inadequacy related to HL and was treated as a distinct construct rather than a measure of stigma. The Pressure-to-be-assessed section reflects perceived pressure from others to seek hearing assessment. Thus, the four selected captured distinct but potentially related psychosocial responses to HL and HA use.

Responses were rated on a three-point Likert scale (1 = Not True, 2 = Partly True, 3 = True). One question in HAsS was scored reversely: “I think the behind-the-ear aids are really quite small and inconspicuous” (1 for “True,” 2 for “Partly True,” and 3 for “Not True”), so that a higher score indicates the specified attitude more strongly. For the HLS, HAsS, and PDI sections item responses were summed to produce a total score, with higher scores indicating stronger endorsement of the corresponding attitude (22). HLS comprised of five items, with a minimum possible score of five and a maximum possible score of 15. Low HLS was considered with scores from 5–8, average 9–11, and high 12–15 points. HAsS, included nine items, with a minimum possible score of nine and a maximum possible score of 27. Low HAsS was considered with scores from 9–12, average 13–15, and high 16–27. The PDI section included nine items, with a minimum possible score of nine and a maximum possible score of 27. Low PDI was considered with scores from 9–16, average 17–22, and high 23–27 (22).

### 2.3 Validity and internal consistency of the instrument

To our knowledge, a previously validated Arabic version of the HARQ was not available; therefore, the selected HARQ subscales were translated into Arabic specifically for the present study. The selected subscales were translated from English to Arabic using the forward–back method. One bilingual author translated the selected subscales from English into Arabic. To assess the accuracy and equivalence of the translation, the Arabic version was independently back-translated into English by another bilingual author. The back-translated version was then compared with the original English version, and discrepancies were reviewed to ensure consistency in meaning. This forward–back translation approach is consistent with established recommendations for the cross-cultural adaptation of self-report measures (23). The translated questionnaire was reviewed by a panel of five experts, including three specialists in audiology and two in health sciences, who provided qualitative feedback regarding the clarity, comprehensibility, and cultural appropriateness of the Arabic items. Their comments were reviewed by the research team and incorporated where appropriate. Item–total correlations were examined to assess internal consistency, with correlation coefficients >0.3 indicating acceptable item contribution to the scale. Reliability was assessed using Cronbach's α for each section scoring. Although item responses used a 3-point format, standard Cronbach's alpha was reported as a conservative lower-bound estimate of internal consistency, which yields stable estimates in sample sizes exceeding 300 (24).

For the HLS subscale (5 items, *n* = 403), internal consistency was excellent (Cronbach's α = 0.92, 95% Confidence interval (CI): 0.916–0.938). Item deletion diagnostics indicated that no item should be deleted; thus, all items were retained. Corrected item-total correlations ranged from *r* = 0.84 to *r* = 0.90, *p* > 0.05 for all items.

For the HAsS subscale (9 items, *n* = 403), internal consistency was very good (Cronbach's α = 0.88, 95% CI: 0.868–0.902). Item deletion diagnostics indicated that no item should be deleted; thus, all items were retained. Corrected item-total correlations ranged from *r* = 0.35 to *r* = 0.85, *p* > 0.05 for all items.

For the Pressure-To-be-Assessed subscale (3 items, *n* = 403), internal consistency was excellent (Cronbach's α = 0.92, 95% CI: 0.914–0.939). Item deletion diagnostics indicated that no item should be deleted; thus, all items were retained. Corrected item-total correlations ranged from *r* = 0.90 to *r* = 0.95, *p* > 0.05 for all items.

For the PDI subscale (9 items, *n* = 403), internal consistency was excellent (Cronbach's α = 0.94, 95% CI: 0.939–0.954). Item deletion diagnostics indicated that no item should be deleted; thus, all items were retained. Corrected item-total correlations ranged from *r* = 0.70 to *r* = 0.89, *p* > 0.05 for all items. Finally, the translated questionnaire was pilot-tested among 40 adults with HL with characteristics similar to the target study population to assess clarity and comprehensibility. No changes to the questionnaire were considered necessary following the pilot. Participants in the pilot study were subsequently included in the final study sample.

### 2.4 Statistical analysis

The data were coded by the researchers, and the analysis was done using the JMP software (version 14.2, Cary, NC: JMP Statistical Discovery LLC). Descriptive statistics are presented in frequency tables. A chi-square test was used to assess the associations between categorical variables such as demographic factors and stigma levels. When small cell counts yielded expected frequencies of < 5 in more than 20% of cells (e.g., HL type classifications), Fisher's exact test was used instead of Pearson's chi-square test to ensure statistical precision. The effect size was investigated for each cross tab according to the level of measurement of the variables involved in each table: Lambda Asymmetric test for tables with nominal and ordinal variables. A multiple linear regression model was used to predict both: HLS and PDI from the collected variables in the studied sample as this method allows for identifying significant predictors while controlling for potential confounders. Multivariable binary logistic regression was utilized to predict using hearing aids when medically indicated. One way ANOVA was used to compare average score of pressure to be assessed in different levels of HLS and Cohen's *d* test was done to investigate the effect size of this relationship. Cohen's *D* test is used to investigate the effect size between groups. A two-way ANOVA test was used to determine if the effect of the level of income on HLS differed across different age groups, since this method is appropriate for testing interaction effects between two independent variables on a continuous outcome. *Post-hoc* pairwise comparisons were conducted using Tukey's Honestly Significant Difference (HSD) test. The effect size of 2-way ANOVA was determined using the Partial Eta Squared. The results were considered non-significant (NS) if the *p*-values >0.05, and significant (S) if the *p*-value < 0.05; the term “highly” significant (HS) was used to denote if the *p*-value < 0.01.

#### 2.4.1 Prespecified vs. exploratory analyses and multiplicity control

The study protocol prespecified four primary confirmatory models: (1) multiple linear regression predicting HLS from demographic covariates, (2) 2-way factorial ANOVA testing the age ^*^ income interaction, (3) multivariable binary logistic regression predicting HA adoption, and (4) regression modeling PDI. All other bivariate comparisons (e.g., cross-tabulations by HL type) were secondary/exploratory. To guard against Type I error inflation, primary hypothesis testing was restricted to prespecified multivariable models, and exact *p*-values, 95% confidence intervals, and effect sizes are reported throughout. Primary statistical conclusions remained robust when evaluated against adjusted alpha thresholds.

### 2.5 Ethical considerations

Ethical approval was secured from the Institutional Review Board committee at Princess Nourah bint Abdulrahman University (#22-0395). Participation was anonymous, voluntary and informed consent was obtained on the questionnaire's first page. Participants were assured that their responses would be confidential and that no personal identifiers would be collected. All data were stored on password-protected devices accessible only to the research team. The study adhered to the ethical principles outlined in the Declaration of Helsinki regarding research involving human participants, including protection of sensitive information and data anonymisation throughout the process.

## 3 Results

Results are presented in the following order: participant characteristics, prevalence of stigma, associations between stigma and HA use, predictors of HLS and personal distress, and additional analyses examining HL type and income–age interactions.

### 3.1 Participant and hearing loss characteristics

Sociodemographic information of the participants is presented in Table 1. The total sample size was 403. The male-to-female ratio was nearly 1:1, reflecting a balanced gender representation. Approximately half of the participants were married. Age distribution revealed that 40% of participants fell within the 18–40 years bracket, while only 13% were 61 years or older. Geographical diversity was evident in the sample, with the majority hailing from the Middle Region (46.15%) and the Western Region (22.83%), collectively representing over two-thirds of the sample. The Northern Region had the smallest representation accounting for 6.20% of participants.

**Table 1:** Sociodemographic criteria of the studied sample (*n* = 403).

Category	*N*	%
Gender		
Female	189	46.90%
Male	214	53.10%
Age (years)		
18–30	100	24.81%
31–40	79	19.60%
41–50	90	22.33%
51–60	81	20.10%
61 or more	53	13.15%
Residence		
South	42	10.42%
East	58	14.39%
North	25	6.20%
West	92	22.83%
Middle	186	46.15%
Educational attainment		
High school or less	112	27.79%
Bachelor's or diploma	238	59.06%
Post-graduate	41	10.17%
Illiterate	12	2.98%
Marital status		
Single	114	28.29%
Married	203	50.37%
Widow	31	7.69%
Divorced	55	13.65%
Working status		
No	203	50.37%
Yes	200	49.63%
Monthly income, SR (approximate USD equivalent)		
< 5,000 (≈1,333 USD) (Low)	52	12.90%
5,000–10,000 SR (≈1,333–2,666 USD)	156	38.71%
11,000–20,000 SR (≈2,933–5,333 USD)	146	36.23%
More than 20,000 SR (≈>5,333 USD)	49	12.16%

^*^SR = Saudi riyal; USD equivalents are approximate, based on 1 USD ≈ 3.75 SAR.

Educational attainment varied among participants, with approximately one-fourth (27.79%) having completed secondary school or less, and the majority (59.06%) holding a bachelor's degree. Regarding employment status, half of the participants (50.3%) were not actively employed at the time of the study. Income distribution revealed that 12.9% of participants reported a monthly income of < 5,000 SAR (≈1,333 USD), while 38.71% reported earning between 5,000 and 10,000 SAR (≈1,333–2,666 USD). Additionally, 36.23% of participants reported an income between 11,000 and 20,000 SAR (≈2,933–5,333 USD), and 12.16% reported earning more than 20,000 SAR (≈>5,333 USD) per month.

Table 2 presents the self-reported hearing characteristics of the study participants. The majority of participants reported sensorineural HL (77.67%), while conductive HL was present among 2.73%, and only 4.96% reported having mixed HL. A small proportion 8.19% reported HL in one ear, whereas the vast majority (91.81%) indicated HL in both ears. Regarding self-reported severity of HL as classified by the World Health Organization (25), approximately half of the participants (52.36%) reported severe to profound HL, while one-third (31.27%) reported moderate HL. Regarding the duration of HL, 8% reported having HL since birth, 39.7% reported being diagnosed within the past 5 years, and 30.52% reported having been diagnosed 10 years or more ago.

**Table 2:** Self-reported hearing loss condition of the studied sample (*n* = 403).

Category	*N*	%
Self-reported type of hearing loss		
Conductive	11	2.73%
Sensorineural	313	77.67%
Mixed	20	4.96%
Not sure	59	14.64%
Affected ear		
One ear	33	8.19%
Both ears	370	91.81%
Self-reported degree of hearing loss		
Mild	66	16.38%
Moderate	126	31.27%
Severe	124	30.77%
Profound	87	21.59%
Method of communication		
Verbal	308	76.81%
Sign language and verbal	80	19.95%
Sign language	13	3.24%
Self-reported duration of hearing loss		
Since birth	34	8.44%
< 1 year	48	11.91%
1–5 years	112	27.79%
6–10 years	86	21.34%
More than 10 years	123	30.52%
Medical indication to use HAs		
No	44	10.92%
Yes	359	89.08%
Use of HAs		
No	85	21.09%
Yes	318	78.91%

Among the participants, 89.08% reported having a medical indication to use a HA as prescribed by their audiologists. However, in terms of actual usage, 78.91% reported using HAs. Additionally, participants were asked about their method of communication with others. It was found that 23.19% of them used sign language with or without verbal communication, while the majority (76.81%) relied solely on verbal communication.

### 3.2 Prevalence of hearing loss stigma and hearing aid stigma

Table 3 presents the prevalence of HLS and HAsS among the study participants. Table 4 shows the association between the use of HAs when medically indicated and both types of stigma. Regarding HAsS, the majority of the sample (82.93%) reported high levels of stigma associated with HAs, while only 9.76% reported a low level. Specific stigmatization beliefs included 40.94% of participants feeling that using a HA would likely make them look stupid, and 23.08% expressing doubts about the effectiveness of HAs. Additionally, half of the sample found it embarrassing to wear HAs, and 47.89% believed that HAs would make them feel aged. A significant portion (59.55%) expressed concerns about how the others would interact with them if they used a HA.

**Table 3:** Hearing aids and hearing loss stigmas present among the studied sample (*n* = 403).

Type of stigma	Variable	True		Partly true		Not true	
		*N*	%	*N*	%	*N*	%
Hearing aid stigma (HAsS)	I think the behind-the-ear aids are quite small and inconspicuous.	80	19.85%	162	40.20%	161	39.95%
	If I wear an aid, people will probably think I'm a bit stupid.	165	40.94%	103	25.56%	135	33.50%
	From what I know, HAs don't help a great deal.	93	23.08%	163	40.45%	147	36.48%
	It would embarrass me to have to wear a hearing aid.	204	50.62%	95	23.57%	104	25.81%
	I think people react differently to you when you are wearing a hearing aid.	226	56.08%	129	32.01%	48	11.91%
	I would stand out in a crowd wearing a hearing aid.	241	59.80%	99	24.57%	63	15.63%
	Many people don't know how to react to you when you have a hearing aid.	240	59.55%	128	31.76%	35	8.68%
	I think that if you wear a hearing aid, people tend to ignore you.	206	51.12%	118	29.28%	79	19.60%
	It would make me feel old to wear a hearing aid.	193	47.89%	85	21.09%	125	31.02%
Hearing loss stigma (HLS)	When you have hearing difficulties, other people ignore you.	222	55.09%	120	29.78%	61	15.14%
	As I see it, I am less of a person because of my hearing difficulty.	173	42.93%	97	24.07%	133	33.00%
	I am sure that some people think I am stupid just because I have a hearing loss.	153	37.97%	93	23.08%	157	38.96%
	I get the feeling that other people find it a strain to talk to me.	216	53.60%	110	27.30%	77	19.11%
	Some people avoid me because of my hearing difficulty.	188	46.65%	112	27.79%	103	25.56%

**Table 4:** A multivariable binary logistic regression analysis to predict hearing aid adoption when medically indicated (*n* = 359).

Predictor	*B*	SE	Wald χ^2^	*p*-value	Adjusted odds ratio (OR)	95% Confidence interval
Intercept (Constant)	3.09	0.86	12.68	< 0.001^**^	–	—
Age (≤ 50 years)	−0.63	0.26	06.09	0.014^*^	0.28	[0.10, 0.77]
Hearing aid stigma	−0.27	0.07	16.21	< 0.001^**^	0.76	[0.67, 0.87]
Hearing loss stigma	0.44	0.09	22.95	< 0.001^**^	1.56	[1.30, 1.87]

^*^p < 0.05.

^**^p < 0.01.

Regarding HLS, more than half of the sample (55.09%) reported feeling ignored by others when experiencing hearing difficulties, while 53.60% believed that others find it burdensome to communicate with them. Furthermore, 46.65% of participants felt that some individuals avoided them because of their hearing difficulties.

### 3.3 Prediction of using hearing aids when medically indicated

Among individuals who were medically indicated to use HAs (*n* = 359), 11.42% did not use them. Interestingly, a substantial portion (82.93%) of those who did not use the HAs, despite medical indication, reported high levels of HAsS. Remarkably, a similar prevalence of high HAsS was observed among HA users (84.91%). A multivariable binary logistic regression analysis was performed to predict HA adoption when medically indicated (*n* = 359). The overall model fit was statistically significant *X*^2^ = 35.5, *p* < 0.01 and *R*^2^ = 0.13 indicating a small effect size. Controlling for other model covariates, age was significantly associated with HA use. Participants aged 50 years or younger had 72% lower odds of using HAs compared to older adults [OR = 0.28, 95% CI (0.10, 0.77)]. Higher HAsS was associated with a significant decrease in device uptake, with each 1-unit increase in HAsS reducing the likelihood of device adoption by 24% among adults [OR = 0.76, 95% CI (0.67, 0.87)]. Conversely, higher HLS was positively associated with HA use, where each 1-unit increase in HLS increased the odds of device adoption by 56% [OR = 1.56, 95% CI (1.30, 1.87)].

Regarding HLS, only 31.27% of the studied sample demonstrated a low level of stigma and 50.12% of them had a high level. HLS was significantly associated with resistance to seeking medical counsel to assess hearing status unless being forced to, as revealed in Table 5. When comparing the mean score for the pressure to be assessed across different levels of HLS it was found that it was significantly lower among those with low HLS and it increased significantly as the level of stigma increased. This association was highly significant (*F* = 173.3, *p* < 0.01). The effect size between groups was investigated by Cohen's *d* test. Specifically, the data showed that Cohen's *d* score was very large between high and low HLS (Cohen's *d* = 2.05) and between high and average HLS (Cohen's *d* = 1.38). However, the effect size was medium between low and average HLS (Cohen's *d* = 0.66).

**Table 5:** Relationship between hearing loss stigma and seeking medical counsel to assess hearing status.

Variables	Level of hearing loss stigma					
	Low (a)		Average (b)		High (c)	
	Mean	Std dev	Mean	Std dev	Mean	Std dev
Average score of being under pressure from others to have a hearing assessment	3.8	1.6	5.0	1.8	7.6	2.0
ANOVA	*F* = 173.3^**^					
*Post hoc*	a-b (*p* < 0.01)^**^					
	b-c (*p* < 0.01)^**^					
	c-a (*p* < 0.01)^**^					
Cohen's *d*	a-b (0.66)					
	b-c (1.38)					
	c-a (2.05)					

^**^p < 0.01.

### 3.4 Predictors of hearing loss stigma and personal distress

Although no significant difference in HLS by gender was observed, and there was no significant difference across regions as regard HLS, HAsS or HA use (*p* > 0.05 for all), several other factors demonstrated significant associations with HLS in this study. HLS was found to be higher among older age groups, participants with lower educational attainment, unmarried participants, those with lower income levels, participants reporting severe degree of HL, and those reporting an HL duration of 5 years or longer. All these factors were included as independent variables in a multiple linear regression model, with the HLS score as the dependent variable (Table 6). The regression model was statistically significant in predicting HLS (*F* = 15.85, *p* < 0.01 for the whole model and *R*^2^ = 0.19, Adj *R*^2^ = 0.18). It was established that these factors can significantly predict HLS (*p* < 0.05 for each and Variance Inflation Factors (VIF) for all predictors were well within acceptable limits (all VIFs < 2). Among these factors, the duration of HL condition and the age exhibited the highest impact on HLS (Std beta coefficients of 0.2 and 0.14, respectively). Beta coefficients indicate the direction and magnitude of the relationship between the dependent (Y) and independent (X) variables. It also represents the rate of change in the dependent variable as the independent variable changes (i.e., which is the increase or decrease in Y for each one unit change in X). The effect size of this association was also assessed by the Adjusted *R*^2^ value for the entire model (Adj*R*^2^ = 0.18). This indicated a small effect size, meaning that 18% of the variation in HLS can be explained by these variables.

**Table 6:** Multiple linear regression to predict hearing loss stigma among the studied sample (*n* = 403).

Term	Beta coefficients	Standardized coefficients (β)	Std error	*t* ratio	Prob>|*t*|	Lower 95%	Upper 95%	VIF
Intercept	11.259644	0	0.192504	58.49	< 0.01^**^	10.88	11.63	–
Age (more than 50)^®^^1^	0.562924	0.14	0.176787	3.18	< 0.01^**^	0.21	0.91	1.04
Education (secondary or less)^®^^2^	0.537198	0.13	0.188373	2.85	< 0.01^**^	0.16	0.90	1.14
Marital status (not married)^®^^3^	0.4391067	0.12	0.169487	2.59	< 0.01^**^	0.10	0.77	1.08
Income (10,000 SR or less)^®^^4^	0.3481971	0.09	0.175687	1.98	0.0482^*^	0.002	0.69	1.16
Self-reported degree of hearing loss (severe)^®^^5^	0.399272	0.11	0.190706	2.09	0.0369^*^	0.02	0.77	1.37
How long have you been diagnosed (more than 5 years) ^®^^6^	0.7449211	0.20	0.186859	3.99	< 0.01^**^	0.37	1.11	1.26
*R*^2^ Adj for the whole model	0.18^**^							

^*^p < 0.05; ^**^p < 0.01.

^®^^1^The reference category of age: 50 years or less.

^®^^2^The reference category of education: College degree or more.

^®^^3^The reference category of marital status: married.

^®^^4^The reference category of income: more than 10,000 SR\month.

^®^^5^The reference category of Self-reported degree of hearing loss: mild.

^®^^6^The reference category of how long have you been diagnosed: 5 years or less.

Table 7 demonstrated that 51.6% of participants reported a high level of PDI. When HLS, HAsS, and self-reported degree of HL were examined as predictors of PDI, only HLS was significantly associated with PDI, whereas HAsS and degree of HL were not significant predictors (*F* = 660.6, *p* < 0.01 for the whole model). The effect size of this association was large as assessed by the Adjusted *R*^2^ value for the entire model (Adj *R*^2^ = 0.83). This indicated that 83% of the variation in PDI can be explained by HLS. To rule out construct contamination and multicollinearity between HLS and PDI, VIF were examined. All VIF values (3.70–3.91) were within acceptable limits (< 5.0). An item-level inspection confirmed that HLS items exclusively measure external/interpersonal social stigma, whereas PDI items measure internal affective distress, confirming structural independence despite the strong empirical correlation (*R*^2^ = 0.83). Sensitivity analysis restricted to univariate modeling confirmed parameter stability (B = 1.36, *p* < 0.001).

**Table 7:** Multiple linear regression model to predict personal distress and inadequacy using both types of stigma and degree of hearing loss.

Term	Unstandardized coefficients (B)	Standardized coefficients (β)	Std error	*t* ratio	Prob>|*t*|	Lower 95%	Upper 95%	VIF
Intercept	4.8	0	0.49	9.87	< 0.01^**^	3.9	5.8	–
Self-reported degree of HL (severe)^®^	0.13	0.02	0.12	1.08	0.2804	−0.10	0.37	1.1
HAsS	0.07	0.06	0.04	1.58	0.1156	−0.01	0.15	3.69
HLS	1.35	0.85	0.06	21.03	< 0.01^**^	1.22	1.48	3.91
*R* ^2^	0.83^**^							
*R*^2^ Adj for the whole model	0.83^**^							

^**^p < 0.01.

^®^The reference category is mild degree HL.

### 3.5 Association between self-reported type of hearing loss, stigma, and hearing aid use

Table 8 presents the association between self-reported type of HL, HLS, and HA use. The majority of the participants reporting conductive HL did not use HAs compared to sensorineural and mixed types (90.9% vs. 10.5% and 35% respectively). This was statistically highly significant (Fisher's exact *p* < 0.01, *n* = 344). On the other hand, the level of HLS showed a significant association with the type of HL. The high level of HLS was not detected among any of the participants reporting conductive type, while 59% of those reporting sensorineural type revealed high HLS vs. 15% of those with mixed type. This was statistically highly significant (Fisher's exact *p* < 0.01, *n* = 344). The effect size of both associations was investigated with Lambda Asymmetric test that was 0.18 and 0.11 respectively which reflects weak association for both.

**Table 8:** Association of the type of hearing loss with hearing loss stigma and hearing aid use.

Category	Type of hearing loss *n* = 344^*^						Test statistic (*p*-value)	Effect size
	Conductive		Sensorineural		Mixed			
	*N*	%	*N*	%	*N*	%		
Use of hearing aids							Fisher's exact *p < * 0.01^**^	Lambda asymmetric = 0.18
No	10	90.91%	33	10.54%	7	35.00%		
Yes	1	9.09%	280	89.46%	13	65.00%		
Hearing loss stigma							Fisher's exact *p < * 0.01^**^	Lambda asymmetric = 0.11
Low	9	81.82%	70	22.36%	12	60.00%		
Average	2	18.18%	56	17.89%	5	25.00%		
High	0	0.00%	187	59.74%	3	15.00%		

^*^59 cases (who did not report the type of hearing loss) have been excluded.

^**^p < 0.01.

### 3.6 Interaction between income and age on stigma

As shown in Figure 2, the level of HLS varied significantly across different age groups according to income level (*p* < 0.002). Notably, in higher income levels, all age groups exhibited nearly the same low HLS scores. However, in lower income levels, older age groups demonstrated higher HLS scores than younger respondents.

**Figure 2:**
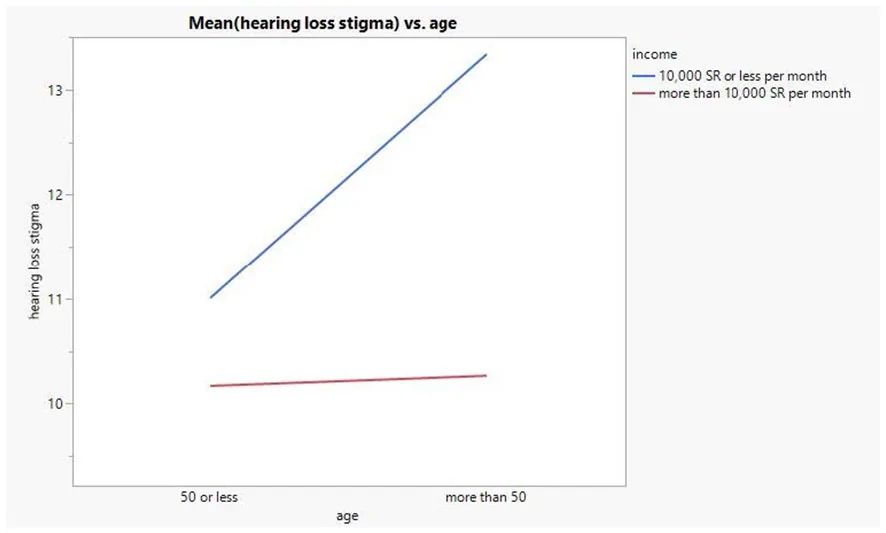
Differences in hearing loss stigma by income level across age groups.

A two-way factorial Analysis of Variance (ANOVA) was conducted to examine the main and interaction effects of age cohort (≤ 50 years vs. < 50 years) and monthly income level (≤ 10,000 SR vs. < 10,000 SR) on HLS (*N* = 403; Table 9).

**Table 9:** Two-Way factorial Analysis of Variance (ANOVA) to examine the main and interaction effects of age and monthly income level on hearing loss stigma (*N* = 403).

Model component/ Subgroup cell	Sample size (*n*)	Estimated marginal mean (LS mean)	Standard error (SE)	95% confidence interval	Tukey group
≤ 50 years, ≤ 10,000 SR/month	134	11.01	0.30	[10.42, 11.59]	B
≤ 50 years, >10,000 SR/month	135	10.17	0.30	[9.59, 10.75]	B
>50 years, ≤ 10,000 SR/month	74	13.34	0.40	[12.55, 14.12]	A
>50 years, >10,000 SR/month	60	10.27	0.44	[9.39, 11.14]	B
**Model parameters**	**Unstandardized coefficient (B)**	**Standard error (SE)**	**Std beta**	***t*** **-statistic**	***p*** **-value**
Intercept	11.20	0.18	0	61.39	< 0.001^**^
Age (< 50 years)	0.61	0.18	0.15	3.33	0.001^**^
Income (≤ 10,000 SR/month)	0.98	0.18	0.27	5.36	< 0.001^**^
Age (< 50) × Income (≤ 10,000 SR)	0.56	0.18	0.15	3.06	0.002^**^

^**^p < 0.01.

Examination of the estimated marginal means (Least Squares Means) revealed that among younger adults (≤ 50 years), HLS scores were relatively comparable between those earning ≤ 10,000 SR\month [*n* = 134, *M* = 11.01, SE = 0.30, 95% CI (10.42, 11.59)] and those earning < 10,000 SR/month (*n* = 135, *M* = 10.17, SE = 0.30, 95% CI (9.59, 10.75). In contrast, among older adults (< 50 years), monthly income exerted a substantial effect on stigma: older adults with lower income (≤ 10,000 SR\month) reported markedly higher stigma scores [*n* = 74, *M* = 13.34, SE = 0.40, 95% CI (12.55, 14.12)] compared to their higher-income counterparts [*n* = 60, *M* = 10.27, SE = 0.44, 95% CI (9.39, 11.14)]. The model revealed a statistically significant 2-way interaction between age cohort and income level, *B* = −0.56, SE = 0.18, *t* = −3.06, *p* = 0.002, 95% CI: −0.92, −0.20. The overall factorial model was statistically significant and accounted for a moderate-to-large portion of the variance in HLS: *F* = 14.93, *p* < 0.0001, accounting for 10.1% of the variance in HLS (*R*^2^ = 0.101, *R*^2^ adj = 0.094, RMSE = 3.44 and the Partial Eta Squared = 0.10).

Further *post hoc* analysis was conducted to probe the significant 2-way age × income interaction. *Post-hoc* pairwise comparisons were conducted using Tukey's Honestly Significant Difference (HSD) test.

As shown in Table 9, Tukey's HSD test revealed that older adults with lower income (< 50 years, ≤ 10,000 SR) reported statistically significantly higher HLS (*M* = 13.34, SE = 0.40) than all other demographic subgroups: older adults with higher income (*M* = 10.27, SE = 0.44; Mean Difference = 3.07, 95% CI (1.53, 4.61), *p* < 0.05), younger lower-income adults (*M* = 11.01, SE = 0.30; Mean Difference = 2.33, 95% CI (1.05, 3.61), *p* < 0.05), and younger higher-income adults (*M* = 10.17, SE = 0.30; Mean Difference = 3.17, 95% CI (1.89, 4.45), *p* < 0.05).

In contrast, *post-hoc* comparisons indicated no statistically significant differences in HLS among the latter three groups (*p* > 0.05), indicating that the older, lower-income subgroup had significantly higher HLS scores than the other three age–income groups.

## 4 Discussion

This study explored stigmatization of HL and HAs use among 403 adults with HL in Saudi Arabia. To our knowledge, little research has specifically investigated HL- and HA-related stigma in the Saudi population. Existing Saudi studies have primarily focused on hearing health awareness rather than stigma. For instance, a study conducted in Jeddah reported only a fair level of public knowledge regarding ear health and HL management, highlighting the need for more comprehensive educational materials and public health initiatives to improve hearing health awareness (26). Increasing public awareness is important because inadequate knowledge may delay the recognition and management of HL and contribute to poorer health and social outcomes. Against this background, the present findings provide important evidence on the extent and correlates of HL- and HA-related stigma among adults with HL in Saudi Arabia.

Younger and middle-aged adults constituted the majority of the study sample, while older adults were less represented. This age distribution should be interpreted in the context of Saudi Arabia's relatively young population, where older adults account for only 3.5% of the population (20). However, because participants were recruited using convenience sampling, the sample cannot be considered representative of the Saudi population. This demographic context should therefore be considered cautiously when comparing the present findings with studies conducted in countries with older populations, such as those in Europe and the United States (27, 28). High levels of HLS and HAsS were reported by 50.12% and 82.93% of participants, respectively, indicating that stigma was common within the study sample. These findings suggest that stigma may represent an important barrier to effective HL management, particularly in relation to HA acceptance and use.

Our findings are consistent with previous research. Although direct comparisons should be interpreted cautiously because cultural contexts, healthcare systems, and study populations differ, similar patterns have been reported internationally (29). For instance, a study of Pakistani young adults with moderate to severe HL who were recommended to use HAs found that 70% of participants who were recommended HAs did not comply with treatment because of concerns about stigmatization (30). Likewise, other studies have shown that a considerable proportion of individuals do not use their HAs regularly because of stigma (31, 32). Furthermore, review studies have consistently demonstrated that both HL and HA use are commonly stigmatized across different populations and settings (17, 33–35).

Although participants were recruited from different regions across Saudi Arabia, no statistically significant regional differences were observed in HLS, HAsS, or HA use (*p* > 0.05 for all). This finding should be interpreted cautiously because the study was not designed to evaluate regional differences in access to hearing healthcare, and the convenience sampling approach may not have captured variation in service availability or utilization across regions. Further research using representative regional samples is needed to determine whether geographic differences influence stigma or hearing-health behaviors in Saudi Arabia.

In the present study, age and duration of HL emerged as the strongest predictors of HLS, with regression estimates of 0.5 and 0.7, respectively. This finding contrasts with previous studies suggesting that younger adults perceive greater stigma because they associate HL with aging (8, 36). In the present sample, older adults and individuals living with HL for longer periods reported higher levels of HLS. One possible explanation is that prolonged exposure to communication difficulties and social challenges may reinforce negative perceptions of HL over time. Similarly, older adults may experience age-related stereotypes that contribute to internalized stigma and reluctance to acknowledge or manage their hearing difficulties (37). Although the difference was not statistically significant, nearly half of the participants reported that wearing HAs made them feel old (Table 5), suggesting that age-related perceptions of HA use may extend beyond older adults.

The self-reported type of HL was also significantly associated with both HA use and HLS (*p* < 0.01). Individuals reporting sensorineural HL were substantially more likely to use HAs (89.46%) than those reporting conductive (9.09%) or mixed HL (65.00%). This finding is consistent with current clinical practice, as conductive HL is often medically or surgically treatable, whereas sensorineural HL is typically permanent and more likely to require amplification (38). Furthermore, sensorineural HL affects not only sound audibility but also sound clarity, frequently resulting in speech perception difficulties, particularly in noisy environments (39). These communication difficulties may contribute to higher levels of perceived stigma through increased social withdrawal, embarrassment, and perceived disability. Consistent with this interpretation, almost 60% of participants reporting sensorineural HL reported high levels of HLS, whereas none of those reporting conductive HL did. Although the effect sizes were small to moderate (Lambda Asymmetric = 0.18 for HA use and 0.11 for HLS), these findings suggest that the relationship between HL type, stigma, and HA use warrants further investigation.

One of the most notable findings of this study was participants' perception of how they are treated by others because of their HL and HA use. A considerable proportion of participants reported feeling that others did not interact with them normally, often describing experiences of being ignored, avoided, or finding communication more difficult. These findings suggest that the behavior of others may contribute to the development and maintenance of perceived stigma among adults with HL. Similar observations have been reported in the broader stigma literature, where ignorance and limited public understanding have been identified as important contributors to stigmatization across a range of health conditions (40, 41). Likewise, previous studies have shown that the general public often has limited knowledge and understanding of HL and HA use (42). Together, these findings suggest that interpersonal experiences and public attitudes may form an important component of HL-related stigma. Encouraging supportive communication within families, friendship networks, and the wider community may help individuals with HL discuss their communication difficulties more openly and enhance their ability to cope with their condition (43).

Another notable finding was the high level of PDI, reported by 51.6% of participants. HLS was strongly associated with PDI, whereas HAsS and self-reported degree of HL were not significantly associated with PDI in the adjusted model. Although HLS and PDI represent distinct constructs, the strong association observed between them suggests that greater HL-related stigma co-occurred with greater personal distress and feelings of inadequacy. This finding is consistent with previous evidence describing negative self-perception, social withdrawal, isolation, shame, and stigma-related identity threat among individuals with HL (18, 19, 44).

Negative perceptions of HAs may persist despite advances in HA technology and reported improvements in user experience and acceptability (45, 46). Ensuring that patients understand these developments may improve adherence to HA use (47). Conversely, failure to address HAsS may reduce communication and negatively affect quality of life. Previous studies have similarly reported greater social isolation among individuals with HL (48), while workplace barriers associated with untreated HL may further limit access to reasonable accommodations and adversely affect employment and career progression (49).

Understanding the factors that influence HA uptake is essential for improving HL management, as stigma has consistently been associated with reduced adherence to HA use (35). Interestingly, the present study found that higher HLS was significantly associated with increased HA use. At first glance, this finding appears counterintuitive, as stigma is generally considered a barrier to rehabilitation. Recent evidence, however, suggests that experiences of HL stigma and responses to stigma, including decisions regarding disclosure of HL, are associated with HA use (50). One possible explanation for our finding is that individuals who perceive greater stigma related to their HL may also be more motivated to adopt HAs to improve communication, reduce the visibility of their disability in social interactions, and regain a sense of confidence and control. This remains a hypothesis, however, as the present study did not examine participants' motivations for HA use or establish the direction of this association. This interpretation is supported by Erler, Garstecki (36), who reported that HA use was associated with confidence and intellect. Consistent with this finding, the present study demonstrated a moderate-to-strong association between HLS and HA use (Gamma = 0.44). This association highlights the potential importance of psychosocial factors in hearing rehabilitation, although the mechanisms and direction of the relationship require further investigation.

In contrast, HAsS was highly prevalent among both HA users and non-users, with 84.91% of HA users and 82.93% of non-users reporting high levels of HAsS. Despite this similar prevalence, the adjusted analysis showed that higher HAsS was associated with lower odds of HA use. This finding is consistent with previous literature identifying stigma and negative perceptions of HAs among the factors that may contribute to HA rejection or non-use (30, 34). At the same time, the high prevalence of HAsS among HA users suggests that some individuals adopt HAs despite continuing to experience negative perceptions surrounding their use. One possible explanation may be the availability of government-supported access to HAs in Saudi Arabia. The Ministry of Human Resources and Social Development provides eligible individuals with hearing disabilities access to assistive devices, including HAs, according to specified eligibility criteria (51, 52). Such support may reduce financial barriers to HA acquisition for eligible individuals, although access should not be assumed to be uniform across the population. Furthermore, health-related decisions are frequently influenced by family members and collective decision-making processes (53). Thus, HLS and HAsS may have different relationships with HA use: higher HAsS was associated with lower odds of use, whereas higher HLS was associated with greater odds of use. However, the mechanisms underlying these relationships were not directly examined and should be regarded as hypotheses requiring further investigation.

Although 11.42% of participants who were medically indicated to use HAs reported not using them, this proportion was lower than that reported in international studies, where only approximately one-third of individuals with HL obtain HAs (54). The reasons for non-use were not explored in the present study but may include financial concerns, limited awareness of government-funded HA services, previous negative experiences, misconceptions, family influences, or persistent stigma (16, 31, 33, 34). Nevertheless, our findings differ from those of Basheer et al. (30), who reported that 70% of participants declined HA use. These contrasting findings may reflect differences in cultural context, healthcare systems, and the relative influence of family and community attitudes on health-related decision-making.

The present study identified several demographic and hearing-related factors associated with higher levels of HLS. Consistent with previous research (55), individuals who reported feeling pressured to undergo a hearing assessment were significantly more likely to experience high levels of HLS. This finding may reflect concerns about being labeled or judged by others, which could discourage timely help-seeking and delay both diagnosis and rehabilitation.

No significant sex differences were observed in HLS, suggesting that HLS may be experienced similarly by males and females within the Saudi population. Although a recent systematic review suggested that sociocultural factors, including stigma, may influence males and females differently, particularly regarding concerns about appearance and HA use (29), the absence of a significant sex difference in the present study suggests that HLS was not strongly differentiated by sex within this sample.

Higher levels of HLS were also associated with lower educational attainment, unmarried status, lower income, greater self-reported HL severity, and a longer duration of HL. These findings suggest that stigma is shaped by both social and hearing-related factors. Individuals with lower educational attainment may have reduced access to health information and a more limited understanding of hearing rehabilitation, whereas those with lower incomes may experience additional social and practical challenges that reinforce negative perceptions of HL. Although government-supported provision of HAs is available to eligible individuals with hearing disabilities in Saudi Arabia (52), the present study did not examine whether educational attainment influenced participants' awareness of, or ability to navigate, available services. Furthermore, participants reporting severe HL reported higher levels of stigma than those reporting milder HL, which is consistent with previous evidence demonstrating poorer quality of life among individuals with more severe hearing impairment (56).

An important observation was the interaction between age and income. While participants with higher incomes generally reported lower levels of HLS across all age groups, older adults with lower incomes demonstrated the highest levels of stigma. Although the present study was not designed to explain this interaction, it suggests that socioeconomic disadvantage may amplify the psychosocial impact of HL in later life. Future longitudinal and qualitative studies are warranted to better understand the mechanisms underlying this relationship and to determine the mechanisms underlying this relationship and its implications for hearing-health support.

These findings also have potential implications for hearing healthcare and public awareness. Consistent with previous literature (35), audiology services may consider incorporating individual and family counseling that addresses stigma, communication strategies, and the benefits of HA use. Public awareness initiatives may also help address misconceptions surrounding HL and HAs, as previous studies have shown that such campaigns can improve awareness of health-related issues (57, 58). In Saudi Arabia, X and television have been identified as preferred platforms for disseminating health information (59) and may therefore provide suitable channels for hearing-health messaging. Such initiatives could be tailored to groups in whom higher HLS was observed in the present study, including older adults and individuals with lower educational attainment or income (60). The association between longer HL duration and higher HLS also suggests that the potential value of addressing stigma earlier in the rehabilitation pathway warrants further investigation. Further research is needed to clarify the mechanisms underlying HLS and HAsS, factors associated with HA uptake, and the effectiveness of interventions aimed at reducing stigma in the Saudi context.

## 5 Strengths and limitations

An important strength of this study is that it addresses a significant gap in the literature by providing evidence on HLS and HAsS among adults with HL in Saudi Arabia, where research on these topics remains limited. The findings provide valuable insights for audiologists into the experiences and needs of adults with HL, enabling more individualized and patient-centered care, particularly for groups at greater risk of experiencing HLS. By recognizing and addressing stigma as part of routine audiological care, clinicians can better support patients in managing their HL, facilitate engagement with rehabilitation, and ultimately improve their quality of life.

While the findings provide important insights into stigma associated with HL and HA use, they should be interpreted within the context of the study's sample characteristics and data collection methods. First, participants were recruited using convenience sampling through social media platforms, which may have underrepresented individuals with limited internet access or lower educational attainment and may therefore limit the generalizability of the findings. Furthermore, the self-presentational nature of social media may have influenced how participants perceived or reported stigma, potentially introducing response bias. As the survey was disseminated across multiple platforms and could be further shared, the total number of individuals reached could not be determined, and therefore a recruitment response rate could not be calculated. Second, hearing status, including the degree, type, and duration of HL, was self-reported rather than confirmed through audiological assessment, introducing the possibility of recall and reporting bias. This limitation may be particularly relevant for age-related HL, which often develops gradually and may be underestimated by affected individuals. In addition, 59 participants were excluded from the HL-type sub-analysis because they were uncertain about their HL type. Although this exclusion was necessary to preserve the validity of the diagnostic categories used in the sub-analysis, it may have introduced selection bias if these participants systematically differed from those who reported a specific HL type in terms of health literacy, access to formal audiological assessment, stigma perceptions, or HA use. Additionally, responses from the small proportion of participants who required assistance with questionnaire completion (2.98%) may have been subject to response bias, as assistance was provided by relatives and was not standardized. Finally, the present study did not specifically validate the psychometric implications of administering only four of the seven HARQ subscales. In addition, although the Arabic translation underwent forward–back translation, qualitative expert review, pilot testing, and internal consistency assessment, a full psychometric validation of the Arabic HARQ, including evaluation of its factor structure and broader construct validity, was not conducted.

## 6 Conclusions

In conclusion, this study provides valuable insights into stigma associated with HL and HA use among adults with HL in Saudi Arabia. High levels of both HLS and HAsS were observed, indicating that stigma was a prominent psychosocial concern within the study sample. HLS was significantly associated with PDI and several demographic and hearing-related factors, including older age, lower educational attainment, unmarried status, lower income, greater HL severity, and longer HL duration. HLS and HAsS showed different associations with HA use; higher HAsS was associated with lower odds of HA use, whereas higher HLS was associated with greater odds of use among individuals for whom HAs were medically indicated. These findings highlight the potential importance of addressing stigma within hearing healthcare and support further investigation of approaches to address HL- and HA-related stigma in the Saudi context.

## Data Availability

The raw data supporting the conclusions of this article will be made available by the authors, without undue reservation.
