# Supplementary material for: Stigma of hearing loss and the use of hearing aids among adults with hearing loss: a cross-sectional study
Source: Front Public Health. 2026 Sep 24;14:1962320. doi: 10.3389/fpubh.2026.1962320 (PMC13646081; doi:10.3389/fpubh.2026.1962320)
Supplement: Supplementary file 1 [file Data_Sheet_1.pdf]

STROBE Checklist – Cross-Sectional Study

Section / topic	Item	Reporting requirement (paraphrased)	Where reported	Status / action
Title and abstract	1a	Identify the study as cross-sectional in the title or abstract.	1	Yes
Title and abstract	1b	Provide an informative, balanced abstract of methods and findings.	1	Yes
Background/rationale	2	Explain the scientific background and rationale.	2–4	Yes
Objectives	3	State the study objectives and any prespecified hypotheses.	4	Yes – objectives stated; no prespecified hypotheses reported.
Study design	4	Present the key elements of the study design early in Methods.	5	Yes
Setting	5	Describe setting/location and relevant dates of data collection/recruitment.	5	Yes
Participants	6	Give eligibility criteria and sources/methods of participant selection.	5–7	Yes
Variables	7	Define outcomes, predictors, confounders/effect modifiers and diagnostic criteria where applicable.	7–8	Yes
Data sources / measurement	8	Describe data sources and measurement methods for variables of interest.	7–8	Yes
Bias	9	Describe efforts to address potential sources of bias.	5, 27–28	Yes
Study size	10	Explain how the study size was determined.	6	Yes.
Quantitative variables	11	Explain handling of quantitative variables and rationale for categorisation.	6–9	Yes
Statistical methods	12a	Describe statistical methods, including adjustment for confounding.	8–9	Yes
Statistical methods	12b	Describe subgroup and interaction analyses.	9, 15	Yes
Statistical methods	12c	Explain how missing data were handled.	6–7	Yes
Statistical methods	12d	Describe methods accounting for the sampling strategy, if applicable.	5, 8–9	N/A / convenience sample – no complex sampling design.
Statistical methods	12e	Describe sensitivity analyses, if performed.	11-15	Yes
Participants	13a	Report participant numbers at each relevant stage.	6, 10; Figure 1	Yes
Participants	13b	Give reasons for non-participation/exclusion at each stage.	27	Yes
Participants	13c	Consider a flow diagram.	Figure 1	Yes
Descriptive data	14a	Report participant characteristics and relevant exposures/confounders.	10–12; Tables 1–2	Yes
Descriptive data	14b	Report numbers with missing data for variables of interest.	Tables 1–2	Yes
Outcome data	15	Report outcome numbers or summary measures.	10–15; Tables 3–8	Yes
Main results	16a	Report unadjusted/adjusted estimates with precision and identify adjusted confounders.	11–15; Tables 4–9	Yes
Main results	16b	Report category boundaries when continuous variables are categorised.	7–8; Tables 1–4	Yes
Main results	16c	Translate relative estimates into absolute risk when relevant.	—	N/A – no relative-risk analysis requiring this translation.
Other analyses	17	Report subgroup, interaction and sensitivity analyses.	11-15; Figure 2	Yes
Key results	18	Summarise key results with reference to study objectives.	16 onward	Yes
Limitations	19	Discuss study limitations, including direction/magnitude of potential bias or imprecision.	27–28	Yes
Interpretation	20	Provide a cautious overall interpretation considering objectives, limitations and other evidence.	16–28	Yes
Generalisability	21	Discuss external validity/generalisability.	27–28	Yes
Funding	22	State funding source and role of funders, or explicitly state no funding.	—	Yes

Source: STROBE cross-sectional reporting checklist, EQUATOR Network / STROBE Initiative.
